# Cerebello-Pontine Angle Tumors in Children: An Update on Challenging Neoplasms

**DOI:** 10.3390/diagnostics16010131

**Published:** 2026-01-01

**Authors:** Luca Massimi, Giuliano Di Monaco, Jacopo Ciccani, Federico Bianchi, Paolo Frassanito, Gianpiero Tamburrini

**Affiliations:** 1 Pediatric Neurosurgery, Fondazione Policlinico Universitario A. Gemelli IRCCS, 00168 Rome, Italy; luca.massimi@policlinicogemelli.it (L.M.); jacopo.ciccani@gmail.com (J.C.); federico.bianchi@policlinicogemelli.it (F.B.); paolo.frassanito@policlinicogemelli.it (P.F.); gianpiero.tamburrini@policlinicogemelli.it (G.T.); 2 Department of Neuroscience, Università Cattolica del Sacro Cuore, 00168 Rome, Italy

**Keywords:** cerebellopontine angle, tumors, ependymoma, schwannoma, intraoperative ultrasounds, AT/RT, neurofibromatosis

## Abstract

**Introduction:** Cerebellopontine angle (CPA) tumors are rare in children. As a result, knowledge on them is still limited, often concerning old series. The goal of this study is to provide an update on these challenging neoplasms by presenting a large series compared with those available in the literature and focusing on tumor characteristics, molecular pattern, extent of tumor removal, surgical complications, and outcome. **Methods:** All children with CPA tumors consecutively operated on between 2010 and 2020 (minimum follow-up: 5 years) and with complete follow-up data were considered. Retro-sigmoid approach was used for tumors arising from CPA (group A) while a midline sub-occipital was used for those extending into CPA (Group B). Intraoperative neuronavigation, neuro-monitoring, and ultrasounds were routinely utilized. **Results:** 48 children (54 tumors) were included (mean age at surgery: 6.9 years, 38% infants, M/F ratio 1.1). Hydrocephalus was present at diagnosis in 27% of cases. Gross total resection of the tumor was obtained in 59% of cases, and subtotal and partial resection in 24% and 17%, respectively. Complications occurred in 25% of cases. Group A was composed of 23 children: the most common tumor was schwannoma (43%) followed by ependymomas, medulloblastoma, AT/RT (13% each), and less common histotypes. Group B was composed of 25 children: ependymomas (60%), AT/RT (20%), medulloblastoma (12%), others (8%). All but one ependymomas belonged to PF-A molecular group, while medulloblastomas were equally divided between WNT and Sonic-Hedgehog. The overall survival rate after a mean 7.2-year follow-up is 71%. A total of 14 patients died because of tumor or disease progression. No statistical differences between the two groups were detected as far as demographic data, tumor growing pattern, extent of tumor removal, complication rate, and overall survival were concerned. Only the mean tumor diameter was significantly longer in group B (3.9 cm vs. 3.3 cm). Apart from some differences in the demography, the extent of tumor removal and complications, no relevant differences were noticed among the series analyzed. **Conclusions:** Pediatric CPA tumors are uncommon but not rare and present significant management challenges. Surgery is demanding. The long-term survival is poorly improved compared with the past and compared with other posterior fossa tumors, the prognosis is mainly related to the biological tumor characteristics and the adjuvant treatments rather than the surgical excision.

## 1. Introduction

Tumors affecting the cerebellopontine angle (CPA) are rare in the pediatric population, representing only 1–3% of all intracranial tumors [1,2], which is different to adults where they account approximately for 5–10% of all intracranial neoplasms [3,4]. CPA can accommodate different types of tumors with distinct histological characteristics. Actually, some neoplasms originate directly from the CPA while other neoplasms arise from nearby structures and subsequently involve the CPA space. In adults, tumors predominantly arise from the CPA and are mainly represented by schwannomas, meningiomas, and epidermoid cysts [5]. In the pediatric population, instead, these histological subtypes are rarely encountered. Indeed, schwannomas are rare and usually associated with neurofibromatosis type 2 while other histotypes arising from CPA (e.g., AT/RT) or tumors extending into the CPA at a later stage, originating from adjacent structures or embryonic remnants (medulloblastoma, ependymoma, astrocytoma), are much more common [1]. The survival rate of CPA tumors in children varies according to the tumor characteristics and, for some of them, to the molecular subgroup. For example, the 5-year overall survival in WNT-pathway medulloblastoma is >90% while it drops to 50% in group 3 [6]. Similarly, PFB ependymoma shows a 5-year overall survival significantly higher (90%) than PFA (67%) [7].

Apart from this general knowledge on posterior fossa tumors, the currently available literature lacks comprehensive data on CPA tumors in children, and only a few outdated articles on purely pediatric case series are available. The present study aims at contributing to the existing knowledge by presenting an updated institutional experience with CPA tumors and comparing that with previous series. The present one is currently the largest available series, consisting of 48 operated children. This comprehensive exploration aims at enriching the current understanding of the intricacies surrounding pediatric CPA tumors.

## 2. Material and Methods

This study is based on a retrospective analysis of all consecutive children operated on for CPA tumors, either primarily arising from this region or involving that secondarily, at a single institution (A. Gemelli Hospital in Rome, Italy). Children coming prevalently from the middle and south part of Italy are referred to this tertiary level center. The last decade was considered, starting from 2010 to 2020, to have a homogeneous population and a minimum 5-year follow-up. Only consecutive patients with complete preoperative and postoperative investigations, pathological data, and complete follow-up data were included.

For lesions strictly confined to the cerebellopontine angle (CPA), a retro-sigmoid approach was used. In younger children, the patient was positioned supine with the head rotated contralaterally, whereas in older pediatric patients, a park-bench position was preferred. In cases of tumors secondarily extending into the CPA, a midline sub-occipital approach was used, with the patient placed in the prone position.

Neuronavigation was utilized in all surgical procedures. Magnetic navigation was preferred in younger patients due to its non-invasive nature, while optical navigation—requiring rigid head fixation using a Mayfield head-holder—was employed in older children. As per our intraoperative protocol, intraoperative ultrasounds (IOUS) were routinely employed in conjunction with neuronavigation, being particularly useful in lesions invading the cerebellar parenchyma. Also, intraoperative neurophysiological monitoring was utilized in all surgeries. The standard protocol included motor evoked potentials (MEPs) and somatosensory evoked potentials (SSEPs) to assess the integrity of motor and sensory pathways of the corticospinal tract, as well as corticobulbar tract monitoring of the ipsilateral facial nerve (VII cranial nerve). Depending on tumor location, additional corticobulbar monitoring was extended to include III, IV, V, and VI cranial nerves as well as the lower cranial nerves (IX, X, XI, and XII cranial nerves). All procedures were performed under the operating microscope (exoscope in the last 5 cases), with endoscopic assistance employed in selected cases—particularly when residual tumor was suspected as reported by others [8].

In every case, a comprehensive histopathological examination of the tumor was carried out, including molecular profiling analyses in more recent cases.

The statistical analysis was carried out through chi-square statistic (using the Fisher exact test) and the Student *t*-test (and by the Wilcoxon signed-rank test, as appropriate). *p* < 0.05 was considered as a statistically significant difference. Ethical approval was waived because of the retrospective nature of the study and all procedures being part of the routine surgical care.

## 3. Results

During the aforementioned period, 723 children were operated on for brain tumors. Among these, 296 patients (41%) harbored tumors located in the posterior cranial fossa. Specifically, 48 of them, with 54 tumors (six of whom presented with bilateral lesions treated individually), presented a CPA neoplasm. This subset represented 14% of posterior fossa tumors and 6% of the overall pediatric brain tumor cohort.

The mean age at surgery was 6.9 years (range: 0.3–18 years). A total of 38% of them were younger than 3 years, 40% fell within the 3- to 11-year age group, while the remaining 21% were older than 11 years. The gender distribution reflected a 1.1 M/F ratio. All patients underwent a minimum 5-year follow-up, with a median follow-up duration of 7.2 years (range: 5–15 years).

According to MRI characteristics, the patients were divided into two groups: group A encompassed children with tumors originating directly within the CPA region (from the cistern of the CP angle, the vestibule-cochlear nerve, and the portion of the cerebellum or the pons delimiting the CP angle) and treated by retro-sigmoid approach (23 patients), while group B included those extending into the CPA from adjacent structures (cerebellar hemisphere, the IV ventricle or the brainstem) and treated by sub-occipital approach (25 patients). The main characteristics of the two groups are summarized in Table 1. Including the bilateral locations, 29 tumors were located in the left CPA, with the remaining 25 cases on the right side. A total of 13 patients presented with hydrocephalus at the time of diagnosis: 3 of them had undergone a ventriculo-peritoneal shunt before referral to our institute; the remaining 10 children underwent perioperative EVD and postoperative ETV (6 cases) or VP shunt (4 cases) according to an Institutional protocol reported elsewhere [9].

One-stage surgery was performed in 43 patients, with 20 cases utilizing the retro-sigmoid approach and 23 cases employing the sub-occipital approach. Conversely, a two-stage surgery was conducted in 5 patients, including 2 cases of ependymomas, 2 cases of AT/RT, and 1 case of schwannoma. Gross total resection (GTR) was achieved in 59% of cases; that is in 32 out of the 54 operated tumors. Subtotal resection (STR), encompassing cases where the residual volume was less than 10% of the initial volume, was achieved in 24% (13 cases) and partial resection (PR) in 17% (9 cases).

Postoperative complications occurred in 12 patients (25%). According to the classification outlined by Sanford et al. [12], 5 patients (10%) experienced major complications, characterized by permanent deficits, namely due to VII/VIII cranial nerve palsy in 2 cases, hemiparesis, VI cranial nerve palsy, and postoperative hemorrhage in one case each. Minor and transient complications were observed in 7 patients (14.5%), involving VII/VIII cranial nerve palsy in 2 cases, low cranial nerve dysfunction in 2 cases, cerebellar dysfunction in 2 cases, and cerebrospinal fluid (CSF) leakage in one case. The distribution of complications in relation to the extent of resection revealed a twofold incidence of major complications compared to minor ones in case of GTR. Instead, STR group exclusively experienced minor complications, while PR group equally exhibited major and minor complications. No surgical mortality was recorded.

Tumor recurrence was observed in 9 cases (18.5%): 4 cases of ependymomas, 3 AT/RT and one case of LGG and medulloblastoma each. At current follow-up, 71% of children are alive. A total of 14 patients (29%) died because of tumor or disease progression: AT/RT (6 cases), ependymomas (3 cases), NF-2 (2 cases), HGG, PNET and medulloblastoma (one case each).

No statistical differences between the two groups were detected as far as demographic data, growing pattern, extent of tumor removal, complication rate, and overall survival were concerned. Only a weak statistical difference (*p* = 0.03) was found about the mean maximal tumor diameter (3.3 vs. 3.9 cm in group A and B, respectively).

## 4. Discussion

The main goal of this paper is to provide an update on CPA tumors, a very rare type of tumors in pediatric neurosurgery. To achieve this goal, the largest and most updated series (to the best of our knowledge) of pediatric CPA tumors is here presented. Actually, due to their rarity, pediatric CPA tumors are not a common object of specific studies or publications (they are usually considered in series including all posterior fossa tumors or mixed series including adults), so specific and updated knowledge on them is lacking. According to the literature, indeed, only four large series are currently available, the last one being published 10 years ago (Table 2): (1) Zuccaro and Sosa detailed their experience on 2007 with a case series of 30 children, presenting 33 CPA lesions, of which 20 originated from the CPA subarachnoid space and 13 from the surrounding structures [13]; (2) in 2009, Tsai et al. published a case series of 29 patients, including 5 with multiple lesions at diagnosis, 8 patients with tumors exclusively outside the CPA, 16 tumors predominantly within the CPA, and 5 arising from the vicinity and primarily growing into the CPA [2]; (3) in 2013, Phi et al. reported on 26 children with a purely CPA tumor [1]; (4) finally, Tomita and Grahovac contributed with a case series encompassing 44 pediatric cases with CPA and cerebello-medullary fissure (CMF) tumors, including 32 cases predominantly in the CPA and/or CMF, and 12 cases extending into the fourth ventricle [14].

### 4.1. Epidemiological Considerations

The data comparison among these series and the present one reveals an increased incidence of CPA tumors than in the past, their rate among all brain tumors ranging from about 2.5% (past series) to 9.7% (Table 2). This rise, rather than to possible environmental changes or intrinsic causes, should be attributed to the wide diffusion of neuroimaging tools and the availability to transfer to tertiary centers (where large series can be collected and published). In other words, such a rise is likely to be part of the general trend of increased numbers of diagnosis of pediatric brain tumors all around the world [15,16].

Similarly, the notable difference in the age at diagnosis, which is currently lower than in the past (Table 2) reflects the overall improvement in diagnostic tools in recent decades, leading to more frequent early diagnoses.

### 4.2. Clinical and Pathological Aspects

Concerning the clinical presentation, both the present study and the Tomita and coworkers’ one [14] show an increased percentage of signs of raised ICP at diagnosis, resulting in a decrease in cases presenting with cranial nerve palsy or cerebellar ataxia. This difference is attributed to the histological variability of the different case series (see Table 1 and Table 3). While the present study and the provided by Tomita and coworkers’ one predominantly showed ependymomas, which are associated with clinical onset featuring raised ICP other than local compression or cranial nerve palsy, the other two studies primarily featured schwannomas, more often associated with cranial nerve disorders.

As pointed out by other authors [1], the present series confirms the increased incidence of malignant phenotypes among CPA tumors (namely, ependymomas and AT/RT), with its obvious prognostic consequences. Such an increased incidence may be explained by the centralization of pediatric brain tumors in tertiary centers and by the improved surgical techniques and technologies which allow the neurosurgeon to face tumors previously considered as not operable. Compared with old series, the newest ones experienced a relative decline of schwannomas incidence and, therefore, of NF-2 cases. This aspect is hard to explain. Actually, if NF-1 is often under-recognized due to its clinical variability (incidence in medical reports is lower than in screening studies), this is unlikely for NF-2 even though no data on its epidemiological trend are available because of its rarity [17]. Once again, the cause of this decline may be found in the different diagnostic and therapeutic options, as early diagnosis, radio-surgical management [18], use of bevacizumab [15] and accurate follow-up in clinics dedicated to rare disease rather than a really epidemiological changes.

### 4.3. Surgical Aspects

The most relevant difference revealed by the present analysis concerns the rate of postoperative complications, significantly higher in the most recent series (56% in the Tomita and coworkers’ series, 61% in the Phi et al. series, 25% in the present one) compared with the past (6%) (Table 2). This occurred in spite of the routine use of refined techniques for intraoperative monitoring in the present and in the most recent series [14]. Several studies actually demonstrated that intraoperative electrophysiological monitoring not only predicts postoperative functional outcomes but also drives safer surgical procedures [19,20,21]. However, it has to be noticed that alternative techniques—such as intraoperative hearing monitoring [22] or preoperative MRI sequences capable of identifying the facial nerve courses [23]—are needed and being investigated to make safer CPA tumor removal. The difference in the complications rate among the series could be mainly attributed to some factors: (1) the not homogeneous assessment of complications across the different studies. Indeed, not all studies distinguished major complications from minor ones. Moreover, as mentioned, the present case series includes a large population of infants where the risk of some complications (e.g., CSF leakage, cranial nerve injury because of narrow surgical corridors and poor cistern spaces) is higher; (2) the unfavorable tumor size and growing pattern (Table 1). Compared with past series, the present one reports also the mean size of the tumor and the growing pattern. The first one was quite relevant (max diameter: 3.3 and 3.9 cm) and, as expected, higher in group B (3.9 cm) where tumors extending into CPA form other regions were included. Both groups, moreover, showed a relatively high rate of tumor with infiltrative pattern (44% and 52%). Both these findings may have contributed to making the surgical resection hard and risky; (3) the extent of surgical resection: the higher the extent of tumor removal, the higher the risk of complications. Indeed, the present and the Tomita and coworkers’ series showed significantly higher rate of GTR (59% and 61%, respectively) compared with the previous 2 series (31% and 36%); (4) the routine use of more precise neuronavigation tools, intraoperative ultrasounds and endoscopy-assisted surgery in the present series may have contributed to the better relationship between extent of tumor removal and complication rate. A further contribution to this balance could have come from the two-stage surgery used in five patients. In these instances (small children with large tumors), indeed, we believe it is preferable to perform the tumor removal in two separate steps (generally, the second step is performed 2 weeks after the first one) to reduce the operating times and the blood losses, thus decreasing the risk of complications and enhancing the chance of GTR.

**Figure 1:**
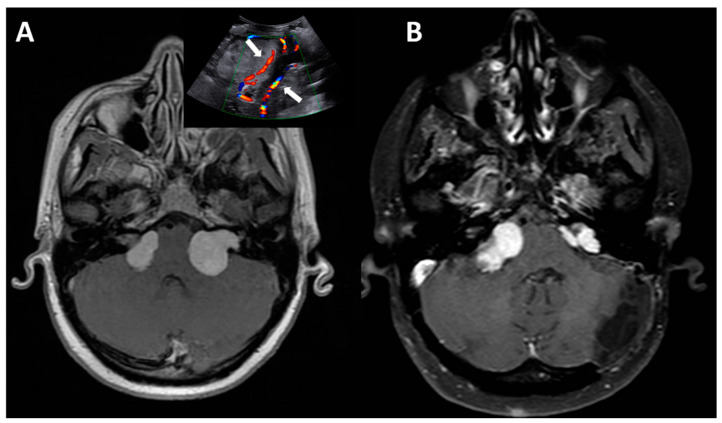
Example of tumor arising directly form the CPA. (**A**) Axial gadolinium MRI showing a bilateral CPA schwannoma in a 14-year-old girl with NF-2. In the box, IUOS demonstrate the relationship of the left tumor with the vertebral arteries (arrows); (**B**) Postop MRI performed 3 months after surgery showing the subtotal removal of the left tumor and the progression of the right one.

**Figure 2:**
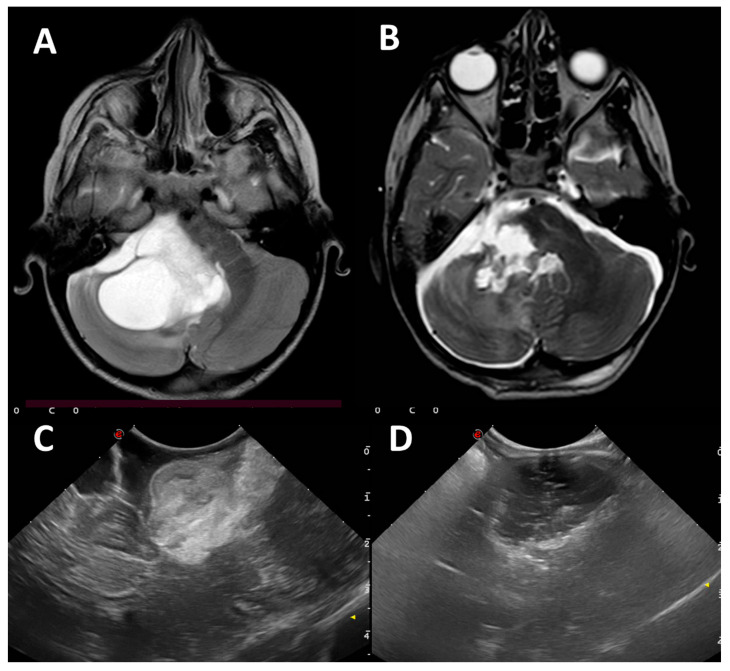
T2 axial MRI showing the preoperative (**A**) and postoperative picture (**B**) of a low grade glioma (9-year-old girl) arising from the brainstem and extending into the right CPA. IOUS clearly show the tumor (**C**) and its GTR (**D**).

**Figure 3:**
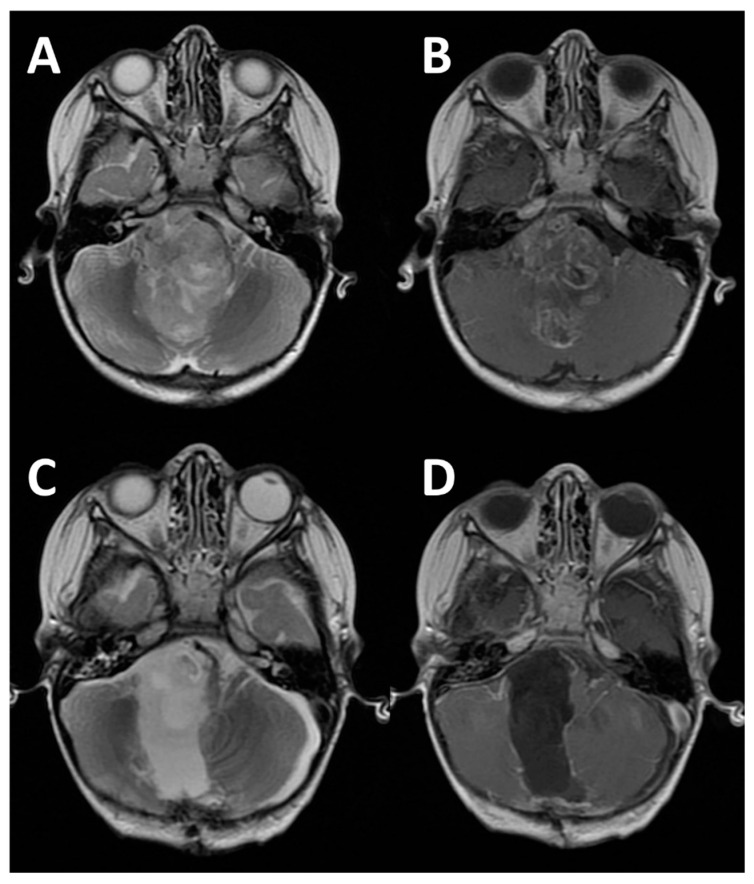
Axial T2 (**A**) and gadolinium MRI (**B**) showing of a 4-year-old boy showing a large PF-A ependymoma obstructing the IV ventricle, occupying large part of the posterior fossa and the right CPA. The same sequences (**C**,**D**) show a GTR after surgery (realized with exoscope).

A further difference with the past concerns the extent of tumor removal. Actually, the rate of GTR is relevantly higher in recent series (59–61%) compared with the previous ones (31–36%), apart from the intermediate (2013) Phi and coworkers’ series (39%) [1], which was characterized by malignant and infiltrative tumors (Table 2). As anticipated, such a difference, at least in part, can result from the high rate of hardly resectable tumors (namely, schwannomas and AT/RT), although the present one shows similar rates of these tumors (Table 3). More likely, the refinement on neuronavigation and intraoperative monitoring techniques, and the more extensive use of IOUS and endoscopy-assisted procedures may have played a role in increasing the GTR rate. In the present series, IOUS played a particularly important role. As previously showed, this simple and reliable tool was of significant help thanks to the possibility to match ultrasounds with MRI-based neuronavigation [24]. This offers the possibility to have a real time navigation (in spite of the brain shift occurring more and more during the operation) and a real time check of the surgical field, aiming at identifying the tumor and its spatial relationships (before tumor removal), checking the tumor and surrounding vessels changes during the removal (Figure 1), and finally looking for possible tumor remnants and intraoperative complications at the end of the removal (Figure 2). In the present series, also the use of exoscope (even if only in the last five cases could have contributed to raising the rate of GTR, including large ependymomas (Figure 3). The exoscope, indeed, has been proven to be particularly useful in pediatric posterior fossa tumors [25]. Similarly, the use of endoscopy-assisted microsurgical resection of CPA tumors has been demonstrated to improve the extent of tumor removal other than to reduce the risk of complication (e.g., facial nerve injury) [26,27]. As experienced in the present series, the endoscope, thanks to the different orientation of the optics, allows to visualize the black angles of the surgical field and the possible tumor remnants. According to the recent literature [28,29,30], the main advantages of endoscopy-assisted microsurgery are the better visualization of the surgical field, the possibility to increase the surgical resection and to reduce the risk of tumor remnants (thus decreasing the risk of tumor recurrence/regrowth), and to reduce the risk of complications. The latter results from a better protection of neurovascular structures and from the missing need to retract the cerebellum to look around the corner. The main limitations of the use of endoscope are related to the risk of injury of the surrounding structures, the lengthening of operating times, and the learning curve. Actually, the endoscope has to be moved into a narrow space (specific manual skills are required, risk of injury of neurovascular structures), without a three-dimensional perception (risk of damage of surrounding structures) and with the need of frequent cleaning of the tip of the endoscope (idem). Thermal damage (in case of xenon source) and need of specific equipment are further limitations. Such disadvantages have limited, so far, the diffusion of this technique even though several authors currently use even a purely endoscopic approach to CPA tumors [31].

### 4.4. Outcome

In terms of OS, the rates remain relatively high either inside the present series (without significant differences between tumors purely arising from and those extended to CPA) (Table 1) and among the other ones (Table 2). Although this outcome is certainly improved compared with the personal experience dated more the 15–20 years ago [32,33,34], it is unchanged across the main series in the last 15 years (Table 2). Such a result is relatively surprising if the recent and continuous improvements in the neurosurgical and neuro-oncological treatments are considered. The possible explanations are as follows: (1) relatively higher number of tumors with benign course (meningiomas, choroid plexus tumors, etc.) or not tumor lesions in some of the past series (Table 3); (2) increased number of malignant tumors (AT/RT, ependymomas) in the most recent ones (Table 3) and with infiltrative pattern (Table 1). As largely experienced [35,36], AT/RT still show a very poor survival rate, accounting for the higher mortality also in the present series. Compared with past series, the present one offers the possibility to analyze the molecular data of most of medulloblastomas and ependymomas treated. As expected, based on the young age and the CPA location [10], almost all the ependymoma cases of the present series belonged to PF-A, thus justifying ependymomas as the second cause of mortality. On the other hand, such a raised risk of mortality was counterbalanced by the good prognosis associated with most of the medulloblastomas due to their low/intermediate risk groups in the present series [11,37]. Finally, the disease progression of NF-2 contributed to the OS equilibrium between the two groups, in spite of the benign biological behavior of schwannomas (Table 1); (3) longer follow-up time in the present series (86.4 months) compared to previous studies (Table 2).

## 5. Limitations

In spite of the largest number of cases and the updates, the study design (case series) remains the main limit of the present study. To date, unfortunately, it is not possible to have different types of study on this topic. However, multicenter and/or perspective studies could be set up in the near future.

## 6. Conclusions

The present study suggests that pediatric CPA tumors are certainly uncommon but not rare and present significant management challenges. Surgery, in particular, is demanding (a complete and advanced surgical armamentarium is required) and risky (still a high rate of complications). Long-term survival is poorly improved compared with the past and compared with other posterior fossa tumors, the prognosis being mainly related to the biological tumor characteristics and the adjuvant treatments rather than the surgical excision.

## Figures and Tables

**Table 1:** Comparison between the two groups.

	Group A	Group B
**No of cases**	23 (28 tumors)	25 (26 tumors)
**M/F ratio**	1.1	1.1
**Mean age at surgery (range)**	8.1 years (0.5–18)	5.7 years (0.3–18)
**Histotypes**	Schwannoma ^: 10 (43%)	Ependymomas °: 15 (60%)
	Ependymomas °: 3 (13%)	AT/RT: 5 (20%)
	Medulloblastoma §: 3 (13%)	Medulloblastoma §: 3 (12%)
	AT/RT: 3 (13%)	Ganglioglioma: 1 (4%)
	Other (4.5% each): epidermoid cyst (1), LGG (1), PNET (1), brainstem astrocytoma (1)	HGG: 1 (4%)
**Main symptoms**	Raised ICP: 9 (39%)	Raised ICP: 18 (72%)
	Cranial nerve palsy: 10 (43%)	Cranial nerve palsy: 5 (20%)
	Cerebellar dysfunction: 2 (9%)	Cerebellar dysfunction: 5 (20%)
	Hemiparesis: 3 (13%)	Hemiparesis: 2 (8%)
	Torticollis: 1 (4%)	Torticollis: 2 (8%)
	Psychomotor delay: 1 (4.5%)	Psychomotor delay: 1 (4%)
**Hydrocephalus at diagnosis**	4 (17%)	9 (36%)
**Mean max diameter**	3.3 cm (range: 1.3–5.5 cm)	3.9 cm (range: 1.5–6.2 cm)
**Growth pattern**	Compression: 13 (56%)	Compression:12 (48%)
	Infiltration: 10 (44%)	Infiltration: 13 (52%)
Extent of resection *	GTR: 14 (50%)	GTR: 18 (69%)
	STR: 9 (32%)	STR: 4 (15%)
	PR: 4 (18%)	PR: 5 (16%)
**Overall survival (deaths)**	74% (6 patients)	68% (8 patients)

* Calculated on 28 tumors for group A and 26 for group B; ^ All but one NF-2 patients. ° Molecular subgroup available in 2/3 cases in group A and 9/15 cases in group B: all PF-A [10] except from one PF-B case (group B). § Molecular subgroup available in 2/3 cases either in group A (one WNT and one Sonic-Hedgehog) and in group B (idem) [11]. AT/RT: atypical teratoid rhabdoid tumor; LGG: low-grade glioma; PNET: primitive neuroectodermal tumors; HGG: high-grade glioma.

**Table 2:** Comparison of the main findings among the 5 series.

	Tsai et al. [2]	Zuccaro et al. [13]	Tomita et al. [14]	Phi et al. [1]	Present Series
N° of cases	29	33	44	26	48
CPA incidence	2.4%	2.6%	n.r.	9.7%	6%
Population age	9.4 y	12.9 y (>11 y)	8.1 y	5.8 y	6.9 y (<3)
Tumor location:					
• CPA • CPA + vicinity	8 21	20 13	32 12	26 -	23 25
Clinical presentation	Ataxia 62% Headache 48% Hemifacial spasm 31% Hemiplegia 31%	Cranial nerve palsy 90% Increased ICP 33% Cerebellar dysfunction 20%	Headache Ataxia Cranial nerve palsy 34%	n.r.	Raised ICP 57% Cranial nerve palsy 31% Cerebellar dysfunction 14%
Predominant Histology	Schwannoma/ glial tumors	Schwannoma	Ependymoma	ATRT/ Schwannoma/Ependymoma	Ependymoma
Surgery	GTR 31% STR 48% PR 7%	GTR 36% STR 54% PR 10%	GTR 62% STR 29% PR 9%	GTR 39% STR/PR 61%	GTR 59% STR 29% PR 12%
Hydrocephalus	44%	33%	52%	46%	27%
Complications	n.r.	2/30	23/44	16/26	12/48
Surgical mortality	2	1	0	2	0
Death FU (range)	10/29 (34%) 38 months (4–225)	7/33 (21%) 61 months (12–144)	13/44 (29%) n.r. (7–180)	8/26 (31%) 38.5 months (0–143)	15/48 (31%) 86.4 months (60–180)

**Table 3:** Histological distribution in different series.

	Tsai et al. [2]	Zuccaro and Sosa [13]	Tomita et al. [14]	Phi et al. [1]	Present Series
Schwannoma	21%	24%	7%	19%	21%
Ependymoma	14%	6%	32%	19%	37.5%
Astrocytomas/LGG	14%	18%	25%	4%	2%
Arachnoid cyst	3%	9%	/	/	/
Medulloblastoma	7%	6%	/	15.4%	12.5%
Glioblastoma/HGG	10%	/	2%	/	2%
Epidermoid cyst	/	6%	9%	4%	2%
ATRT	/	/	9%	19%	17%
PNET	/	3%	7%	8%	2%
Ganglioglioma	/	/	2%	/	2%
Craniopharyngioma	/	3%	/	/	/
Malignant glioneural tumor	/	3%	/	/	/
Meningioma	7%	18%	3%	4%	/
Choroid plexus papilloma	7%	/	/	/	/
Immature teratoma	/	3%	/	/	/
Lymphoma	3%	/	/	/	/
Brainstem glioma	14%	/	/	/	2%
Other Sarcoma	/	/	/	4%	/

## Data Availability

The original contributions presented in this study are included in the article. Further inquiries can be directed to the corresponding author.

## References

[1] Phi J.H., Wang K.-C., Kim I.-O., Cheon J.-E., Choi J.W., Cho B.-K., Kim S.-K. (2013). Tumors in the Cerebellopontine Angle in Children: Warning of a High Probability of Malignancy. J. Neurooncol..

[2] Tsai M.H., Wong A.M.-C., Jaing T.-H., Wang H.-S., Hsueh C., Wu C.-T. (2009). Treatment of Cerebellopontine Angle Tumors in Children: A Single Institution’s Experience. J. Pediatr. Hematol. Oncol..

[3] Grey P.L., Moffat D.A., Hardy D.G. (1996). Surgical Results in Unusual Cerebellopontine Angle Tumours. Clin. Otolaryngol. Allied Sci..

[4] Springborg J.B., Poulsgaard L., Thomsen J. (2008). Nonvestibular Schwannoma Tumors in the Cerebellopontine Angle: A Structured Approach and Management Guidelines. Skull Base Off. J. N. Am. Skull Base Soc. Al.

[5] Samii M., Gerganov V.M. (2012). Tumors of the Cerebellopontine Angle. Handb. Clin. Neurol..

[6] Pan Z., Bao J., Wei S. (2025). Advancing Medulloblastoma Therapy: Strategies and Survival Insights. Clin. Exp. Med..

[7] Zapotocky M., Beera K., Adamski J., Laperierre N., Guger S., Janzen L., Lassaletta A., Figueiredo Nobre L., Bartels U., Tabori U. (2019). Survival and Functional Outcomes of Molecularly Defined Childhood Posterior Fossa Ependymoma: Cure at a Cost. Cancer.

[8] Abolfotoh M., Bi W.L., Hong C.-K., Almefty K.K., Boskovitz A., Dunn I.F., Al-Mefty O. (2015). The Combined Microscopic-Endoscopic Technique for Radical Resection of Cerebellopontine Angle Tumors. J. Neurosurg..

[9] Tamburrini G., Massimi L., Caldarelli M., Di Rocco C. (2008). Antibiotic Impregnated External Ventricular Drainage and Third Ventriculostomy in the Management of Hydrocephalus Associated with Posterior Cranial Fossa Tumours. Acta Neurochir..

[10] Kresbach C., Neyazi S., Schüller U. (2022). Updates in the Classification of Ependymal Neoplasms: The 2021 WHO Classification and Beyond. Brain Pathol..

[11] Juraschka K., Taylor M.D. (2019). Medulloblastoma in the Age of Molecular Subgroups: A Review. J. Neurosurg. Pediatr..

[12] Sanford R.A., Merchant T.E., Zwienenberg-Lee M., Kun L.E., Boop F.A. (2009). Advances in Surgical Techniques for Resection of Childhood Cerebellopontine Angle Ependymomas Are Key to Survival. Childs Nerv. Syst..

[13] Zúccaro G., Sosa F. (2007). Cerebellopontine Angle Lesions in Children. Childs Nerv. Syst..

[14] Tomita T., Grahovac G. (2015). Cerebellopontine Angle Tumors in Infants and Children. Childs Nerv. Syst..

[15] Lu V.M., Elarjani T., Niazi T.N. (2023). Global, Regional, and National Incidence and Mortality Trends in Pediatric Central Nervous System Tumors over the Past 2 Decades. World Neurosurg..

[16] Ostrom Q.T., Cioffi G., Waite K., Kruchko C., Barnholtz-Sloan J.S. (2021). CBTRUS Statistical Report: Primary Brain and Other Central Nervous System Tumors Diagnosed in the United States in 2014–2018. Neuro-Oncology.

[17] Lee T.-S.J., Chopra M., Kim R.H., Parkin P.C., Barnett-Tapia C. (2023). Incidence and Prevalence of Neurofibromatosis Type 1 and 2: A Systematic Review and Meta-Analysis. Orphanet J. Rare Dis..

[18] Kruyt I.J., Verheul J.B., Hanssens P.E.J., Kunst H.P.M. (2018). Gamma Knife Radiosurgery for Treatment of Growing Vestibular Schwannomas in Patients with Neurofibromatosis Type 2: A Matched Cohort Study with Sporadic Vestibular Schwannomas. J. Neurosurg..

[19] Della Pepa G.M., Stifano V., D’Alessandris Q.G., Menna G., Burattini B., Di Domenico M., Izzo A., D’Ercole M., Lauretti L., Olivi A. (2022). Intraoperative Corticobulbar Motor Evoked Potential in Cerebellopontine Angle Surgery: A Clinically Meaningful Tool to Predict Early and Late Facial Nerve Recovery. Neurosurgery.

[20] Hiruta R., Sato T., Itakura T., Fujii M., Sakuma J., Bakhit M., Kojima T., Ichikawa M., Iwatate K., Saito K. (2021). Intraoperative Transcranial Facial Motor Evoked Potential Monitoring in Surgery of Cerebellopontine Angle Tumors Predicts Early and Late Postoperative Facial Nerve Function. Clin. Neurophysiol. Off. J. Int. Fed. Clin. Neurophysiol..

[21] Khan M.M., Dutta A., Rajappa D., Mallik D., Baldoncini M., Rangel C.C., Chaurasia B. (2024). Facial Nerve Electrical Motor Evoked Potential in Cerebellopontine Angle Tumors for Its Anatomical and Functional Preservation. Surg. Neurol. Int..

[22] Miyazaki H., Caye-Thomasen P. (2018). Intraoperative Auditory System Monitoring. Adv. Otorhinolaryngol..

[23] Szmuda T., Słoniewski P., Ali S., Pereira P.M.G., Pacholski M., Timemy F., Sabisz A., Szurowska E., Kierońska S. (2020). Reliability of Diffusion Tensor Tractography of Facial Nerve in Cerebello-Pontine Angle Tumours. Neurol. Neurochir. Pol..

[24] Frassanito P., Stifano V., Bianchi F., Tamburrini G., Massimi L. (2023). Enhancing the Reliability of Intraoperative Ultrasound in Pediatric Space-Occupying Brain Lesions. Diagnostics.

[25] Trezza A., de Laurentis C., Carrabba G.G., Massimino M., Biassoni V., Doro A., Vimercati C., Giussani C.G. (2024). Exoscopic Microneurosurgery in Pediatric Brain Tumors: An Ideal Tool for Complex and Peculiar Anatomo-Topographic Scenarios?. Childs Nerv. Syst..

[26] Raza-Knight S., Chiuta S., Golash A., Gurusinghe N., Roberts G., Alalade A.F. (2022). The Role of Endoscopy in the Resection of Sporadic Vestibular Schwannomas: A Systematic Review of Surgical Outcomes. Otol. Neurotol..

[27] Shrivastava A., Mishra R., Nair A., Nair S. (2021). Endoscopic-Assisted Microsurgery for Vestibular Schwannomas: Operative Nuances. Neurol. India.

[28] De Marco R., Froelich S., Albera A., Garbossa D., Zenga F. (2025). A Systematic Review on the Role of the Endoscope in the Surgical Management of Cerebellopontine Angle Tumors: Is It Time to Draw the Conclusion?. Eur. Arch. Oto-Rhino-Laryngol..

[29] Ruzevick J., Cardinal T., Pangal D.J., Bove I., Strickland B., Zada G. (2023). From White to Blue Light: Evolution of Endoscope-Assisted Intracranial Tumor Neurosurgery and Expansion to Intraaxial Tumors. J. Neurosurg..

[30] Vernon V., Naik H., Guha A. (2022). Surgical Management of Cerebellopontine Angle Epidermoid Cysts: An Institutional Experience of 10 Years. Br. J. Neurosurg..

[31] Gomes F.C., Ferreira M.Y., Pereira M.A.O.M., Ritossa L.A.S., Müller G.C., Cardoso L.J.C., Reis R.D.F., Correa E.M., Ruella M., Champagne P.-O. (2025). Fully Endoscopic Approach to Cerebellopontine Angle Tumors: A Systematic Review and Meta-Analysis. Neurosurg. Rev..

[32] Di Rocco C., Caldarelli M. (1993). General Overview of Posterior Fossa Tumors and Surgical Treatment. Rays.

[33] Di Rocco C., Ceddia A., Iannelli A. (1993). Intracranial Tumours in the First Year of Life. A Report on 51 Cases. Acta Neurochir..

[34] Tamburrini G., D’Ercole M., Pettorini B.L., Caldarelli M., Massimi L., Di Rocco C. (2009). Survival Following Treatment for Intracranial Ependymoma: A Review. Childs Nerv. Syst..

[35] Gupta N.K., Godbole N., Sanmugananthan P., Gunda S., Kasula V., Baggett M., Gajjar A., Kouam R.W., D’Amico R., Rodgers S. (2024). Management of Atypical Teratoid/Rhabdoid Tumors in the Pediatric Population: A Systematic Review and Meta-Analysis. World Neurosurg..

[36] Huang M.A., Margol A. (2025). Current Advances in the Management of Atypical Teratoid Rhabdoid Tumors (ATRT). Adv. Cancer Res..

[37] Thompson E.M., Hielscher T., Bouffet E., Remke M., Luu B., Gururangan S., McLendon R.E., Bigner D.D., Lipp E.S., Perreault S. (2016). Prognostic Value of Medulloblastoma Extent of Resection after Accounting for Molecular Subgroup: A Retrospective Integrated Clinical and Molecular Analysis. Lancet Oncol..

